# Immune Cell Number, Phenotype, and Function in the Elderly with Sepsis

**DOI:** 10.14336/AD.2020.0627

**Published:** 2021-02-01

**Authors:** Wanxue He, Kun Xiao, Min Fang, Lixin Xie

**Affiliations:** ^1^College of Pulmonary and Critical Care Medicine, Chinese PLA General Hospital, Beijing, China.; ^2^Institute of Microbiology, Chinese Academy of Sciences, Beijing, China.

**Keywords:** sepsis, aging, immunosenescence, immune cell, dysfunction

## Abstract

Sepsis is a form of life-threatening organ dysfunction caused by dysregulated host responses to an infection that can be partly attributed to immune dysfunction. Although sepsis affects patients of all ages, elderly individuals display increased susceptibility and mortality. This is partly due to immunosenescence, a decline in normal immune system function associated with physiological aging that affects almost all cell types in the innate and adaptive immune systems. In elderly patients with sepsis, these alterations in immune cells such as endothelial cells, neutrophils, monocytes, macrophages, natural killer cells, dendritic cells, T lymphocytes, and B lymphocytes, are largely responsible for their poor prognosis and increased mortality. Here, we review recent studies investigating the events affecting both innate and adaptive immune cells in elderly mice and patients with sepsis, including alterations in their number, phenotype, and function, to shed light on possible new therapeutic strategies.

Increased global life expectancy coupled with a decrease in birth rates has led to an extraordinary demographic revolution characterized by an explosive numerical and proportional increase in the elderly population [1]. In fact, there were a reported 841 million people over the age of 60 worldwide in 2013, four times higher than the 202 million elderly individuals alive in 1950, and it is expected that this population will surpass two billion by 2050 [2]. Elderly individuals are more susceptible to infectious diseases and display a worse prognosis and higher mortality, partly due to the decrease in immune responses that occurs with aging, known as immunosenescence [3].

Sepsis is defined as “life-threatening organ dysfunction caused by a dysregulated host response to an infection” by the Third International Consensus Definitions for Sepsis and Septic Shock (Sepsis-3), and is one of the most life-threatening critical infectious illnesses [4]. A deeper understanding of the definition of sepsis and progress in resuscitative strategies has shown that immune imbalance is a key pathophysiological change in septic patients that affects almost all cells in both the innate and adaptive immune systems [5].

## Differences between adult and elderly patients with sepsis

Although sepsis can affect patients of all ages, elderly patients suffering from sepsis present with extraordinary and distinct features compared to their younger counterparts. These differences between adult and elderly patients with sepsis include incidence, diagnosis, mortality and prognosis, and susceptibility.

### Incidence

Many studies have indicated that the incidence of sepsis increases markedly with age. For instance, Martin et al. observed that the cumulative age-specific incidence of sepsis increased exponentially across all age deciles and that the relative risk of sepsis was 13.1 times higher in elderly patients than in adult patients [6]. Similarity, Angus et al. reported that the incidence of severe sepsis increased significantly with age, from 0.53 % in 60 to 64 year-old patients to 2.62 % in those over 85 years old [7]. Thus, the incidence of sepsis increases remarkably with age.

### Diagnostic challenges

In the elderly, sepsis is challenging to diagnose and is likely to be missed if not anticipated since both infection and organ dysfunction present differently in these patients. Older adults suffering from infections often present with atypical, nonspecific symptoms and the initial signs of a systemic inflammatory response, such as fever, may be blunted. Indeed, it has been reported that approximately 20-30 % of elderly patients suffering from infections present with a blunted or entirely absent fever response [8]. This may be due to the fact that older adults have a lower basal body temperature and display reduced temperature responses to infections as the hypothalamus is less sensitive to cytokines, leading to poor thermoregulation [9, 10]. In addition, elderly patients exhibit several nonspecific clinical manifestations of infection, including altered mental status, lethargy, weakness, dizziness, tachypnea, loss of appetite, and malaise [11-13]. Diagnosis is also made more challenging by baseline elevations in biomarkers such as CRP, lactate, IL-6, and procalcitonin due to increased age and the presence of multiple disease states [14]. Furthermore, a lack of cooperation among elderly patients who are frail, dehydrated, debilitated, and cognitively impaired can make it difficult to take adequate specimens [13, 15]. Together, these factors make it difficult to correctly and promptly diagnose sepsis in the elderly, often resulting in the optimum timing for treatment being missed.

### Mortality and prognosis

Sepsis fatality has been shown to increase linearly with age deciles with an average of 27.7 % for elderly patients versus 17.7 % for adult patients, demonstrating that age is an independent predictor of mortality in sepsis [6]. Remarkably, elderly non-survivors have been found to die earlier during hospitalization, while elderly survivors are more likely to require health care after hospitalization and display higher comorbidity rates, which affect long-term survival after sepsis [16]. Carbajal et al. observed that 9 % of all sepsis patients discharged from hospital globally died within the two-year follow-up period; however, this rate reached 20 % among the elderly [17]. Similarity, Lemay et al. reported that approximately half of elderly sepsis survivors die within two years of hospital discharge [18]. Therefore, elderly patients with sepsis exhibit poor prognosis and increased mortality, which places an increased burden on global medical resources.

### Susceptibility

The evidence presented above demonstrates that elderly patients with sepsis experience increased incidence, atypical symptoms, higher mortality, and poor prognosis, which can all be caused by a variety of issues, including malnutrition, anatomical and physiological modifications, coagulation abnormalities, age-associated comorbidities, and immunosenescence [19, 20]. Immunosenescence generally refers to age-related immune system dysfunction and is the combined result of all parts of the host defense system, suggesting the involvement of many different cells from both the innate and adaptive immune systems [3]. Thus, immunosenescence plays a prominent role in increasing the susceptibility of older patients to sepsis and their poor prognosis [20, 21].

It is therefore important to identify the mechanisms underlying immune imbalance in elderly patients with sepsis and to develop tailored treatments that improve their prognosis and reduce mortality. Here, we review recent studies that investigate changes in the immune cells of both the innate and adaptive immune systems of elderly mice and patients with sepsis, including alterations in their number, phenotype, and function, which might shed light on possible new therapeutic strategies.

## Numerical, phenotypic, and functional alterations in innate immune cells and potential therapeutic targets

Although innate immune responses are nonspecific, the innate immune system acts as the first line of defense against pathogens and responds rapidly to stimuli. In addition, innate immune cells play important roles in initiating effective adaptive immune responses. Sepsis and aging cause significant alterations in innate immune cells, including numerical, phenotypic, and functional alterations. Here, we review these alterations and the underlying mechanisms in neutrophils, monocytes, macrophages, natural killer (NK) cells, dendritic cells (DCs), and endothelial cells (ECs).

### Neutrophils

Neutrophils are the most prevalent cell type in the innate immune system where they act as first responders to infections, thus are essential for microbial containment and eradication [22]. Numerous studies have suggested that neutrophils may act as antigen-presenting cells and as a channel of communication between innate and adaptive responses by activating T cells [23]. In addition, a novel mechanism has recently been revealed via which neutrophils eliminate various pathogens by forming neutrophil extracellular traps (NETs), which involve the release of antimicrobial proteins anchored to a chromatin network of activated neutrophils [24]. Thus, it is unsurprising that neutrophils play key roles in the defense against infections such as sepsis.

During sepsis, circulating neutrophils present with phenotypic and functional alterations. For instance, Demaret et al. observed that patients with septic shock harbored an increased proportion of immature CD10^dim^ CD16^dim^ granulocytes, which appeared as banded and hypersegmented nuclear cells [25]. These immature neutrophils not only differ in phenotype and morphology, but also in function, with Drifte et al. reporting that circulating immature neutrophils from septic patients are less capable of defending pathogens than mature neutrophils [26]. Several significant functional abnormalities have been investigated in neutrophils. One of the most striking changes during sepsis is the delayed neutrophil apoptosis, unlike the accelerated apoptosis observed in lymphocytes, which leads to ongoing neutrophil dysfunction when combined with an increase in immature neutrophils [27, 28]. Demaret et al. also observed significantly reduced oxidative burst and decreased intracellular myeloperoxidase and lactoferrin expression in neutrophils during sepsis, leading to the impaired production of bactericidal agents. In addition, neutrophils display a reduced chemotactic response during sepsis, which may be due to decreased CXCR1 and CXCR2 expression [25]. Other studies have reported the presence of an immunosuppressive neutrophil subset during sepsis, as characterized by the increased production of large amounts of the immunosuppressive cytokine IL-10 and further suppression of T cell proliferation and function [29, 30]. Although NET formation is generally beneficial for pathogen clearance, excessive NETs can lead to severe tissue damage associated with sepsis severity and organ dysfunction [31]. Together, these phenotypic and functional changes in neutrophils during sepsis reduce bacterial clearance and make septic patients more susceptible to nosocomial infections, leading to poor prognosis.

Although the number of neutrophils is preserved well in elderly individuals, their chemotactic, phagocytic, and NET formation abilities are impaired with age [32, 33]. However, few studies have examined numerical and functional alterations in neutrophils in elderly animals and patients with sepsis. Nacionales et al. observed that elderly septic mice display reduced neutrophil recruitment, phagocytosis, and chemotaxis than their younger counterparts, as demonstrated at the transcriptome level [34]. In addition, the reduced migratory accuracy of neutrophils combined with impaired efferocytosis and blunted responses have been found to lead to a prolonged period of immune suppression, partly resulting in higher mortality in elderly patients and animals with sepsis [35]. Nevertheless, further studies are required to investigate numerical, phenotypic, and functional alterations in neutrophils in elderly septic patients.

Granulocyte colony-stimulating factor (G-CSF) is a glycoprotein that stimulates the production of stem cells and granulocytes, and can increase neutrophil number and function [36]. Two randomized clinical trials have been conducted with recombinant G-CSF to increase neutrophil production, maturity, and function in sepsis [28]. Since neutrophil dysfunction has been observed in elderly patients with sepsis, including reduced recruitment and impaired phagocytosis and chemotaxis, future clinical trials may show that G-CSF administration improves neutrophil function, eradicates infection, and increases the survival of elderly patients with sepsis [35].

### Monocytes and macrophages

The majority of tissue macrophages are derived from monocytes in the peripheral blood as a result of inducible factors in the microenvironment. Monocytes and macrophages are important in the response to pathogens due to their ability to initiate and sustain innate immune responses via phagocytosis and cytokine release [37]. In addition, they can present antigens to adaptive immune cells and amplify anti-pathogen immune responses [38].

The effect of sepsis on the phenotype and function of monocyte subpopulations has been investigated intensely over the past few decades. The most prominent feature of sepsis-induced immunosuppression is likely the diminished capacity of monocytes and macrophages to respond to subsequent challenge with LPS or other inflammatory stimuli, which illustrates the phenomenon of endotoxin tolerance [39]. One effect of endotoxin tolerance on monocytes and macrophages is the reduced release of proinflammatory cytokines, such as TNF-α, IL-1α, IL-6, and IL-12, alongside the enhanced release of anti-inflammatory mediators, such as IL-10. These changes have been demonstrated by analyzing the mRNA of monocytes isolated from patients with sepsis, which display increased expression of inhibitory cytokine genes and decreased expression of proinflammatory molecule genes [40-43]. In addition, endotoxin tolerance reduces the expression of HLA-DR, a known marker of monocyte anergy [44], which is characterized by PD-L1 overexpression and an increased percentage of monocytic myeloid-derived suppressor cells (MDSCs, CD14^+^CD15^-^HLA-DR^-^ cells), leading to impaired antigen presentation and suppressed T cell responses [45, 46]. These alterations have been shown to clearly correlate with hospital-acquired, ongoing, and secondary infections, which ultimately increase sepsis-associated mortality [38].

Although macrophages display significant age-related functional alterations, their numbers remain constant. In elderly individuals, the initial response of macrophages to microbes and other inflammatory stimuli is reduced, likely as a result of diminished TLR expression and associated downstream signaling caused by aging [47]. Van Duin et al. observed that macrophages isolated from elderly mice show diminished responses to TLR-1, TLR-2, and TLR-4 stimulation and reduced TNF-α, IL-1β, and IL-6 production due to attenuated activation of proinflammatory signal transduction in the NF-κB, p38, and JNK pathways [48-50]. In addition, Li et al. elucidated the underlying mechanism via which aging impairs macrophages phagocytosis. They found that alveolar macrophages isolated from elderly mice displayed decreased Rac1 mRNA expression, resulting in decreased Rac1-GTP levels and Arp2/3 activation, attenuated downstream F-actin polymerization, filopodia formation, and reduced cell surface expression of MARCO, which is important for macrophages phagocytosis [51]. Moreover, the ability of macrophages to present antigens to CD4^+^T cells was shown to decline due to decreased HLA-DR expression during aging [52], while their ability to clear apoptotic cells, known as efferocytosis, was also found to decline with age, increasing their inability to effectively clear infections and leading to excessive inflammation and tissue damage [53, 54].

Despite these findings, few studies have focused on the number and function of monocytes and macrophages in elderly patients and animals with sepsis. Rondina et al. observed that platelet-monocyte aggregate (PMA) formation was enhanced in elderly patients with sepsis alongside increased IL-6 and IL-8 and proinflammatory monokines synthesized by monocytes, both of which correlated significantly and positively with 28-day mortality [55]. In addition, enhanced platelet-monocyte aggregation has been shown to amplify inflammatory and thrombotic responses in elderly septic patients, potentially contributing toward impaired immune responses and excess risk of organ failure, disability, and death [13]. Consequently, further studies are urgently needed to determine the role of monocytes and macrophages in elderly patients with sepsis.

Both IFN-γ and granulocyte-macrophage colony-stimulating factor (GM-CSF) are major activators of monocytes and macrophages [39]. In patients with sepsis, treatment with recombinant IFN-γ or GM-CSF has been shown to increase phagocytosis and HLA-DR expression in monocytes and macrophages, while improving survival [56, 57]. Since both aging and sepsis can reduce HLA-DR expression, antigen presentation, phagocytosis in monocytes and macrophages, which may cause additive effects on heightened suppressive function in the elderly septic patients, IFN-γ and GM-CSF could be therapeutic alternatives to reverse monocyte and macrophage dysfunction in elderly septic patients to improve survival.

### NK cells

NK cells are a cohort of lymphocytes known as innate lymphoid cells that play important roles in initiating host defenses and coordinating innate and adaptive immune responses [58]. In addition to their ability to mount a rapid, nonspecific innate immune response against cells infected with intracellular pathogens, such as viruses[59], NK cells can rapidly migrate to the site of bacterial infection and coordinate early responses by amplifying the antimicrobial functions of myeloid cells, particularly macrophages, by releasing IFN-γ [60]. NK cells in human peripheral blood can be divided into two distinct phenotypic subsets based on their surface expression of the homophilic binding glycoprotein CD56: CD56^bright^ and CD56^dim^, which represent cytokine production and cytotoxicity, respectively [61].

During sepsis, NK cells undergo numerical, phenotypic, and functional alterations. Patients with sepsis display a marked decreased in the number of circulating NK cells, which is associated with increased mortality, potentially due to increased apoptosis [62]. In addition, studies have indicated that sepsis affects both CD56^bright^ and CD56^dim^ NK cell subsets; for instance, Forel et al. observed that NK cell functions such as cytotoxicity and cytokine secretion (i.e. IFN-γ) are significantly reduced in septic mice and patients [63]. Similarity, the expression of NKG2D, which is involved in the recognition of pathogen-induced cell surface molecules, is lower in septic patients than in non-septic patients and may reduce cytotoxicity [64]. Jensen et al. also demonstrated that the sepsis-induced impairment of intrinsic NK cell function is associated with reduced DAP12 adaptor protein expression and clustering, followed by diminished AKT phosphorylation and calcium flux capacity upon stimulation [62]. It has been shown that these persistent NK cells functional impairments are closely related to sepsis-induced immunosuppression, making patients more susceptible to secondary infections or latent viral reactivation and leading to poorer outcomes [65, 66]. Nevertheless, several studies have suggested that NK cells could be excessively activated during sepsis, contributing toward the amplification of systemic inflammation. Excessive activation is followed by excessive IFN-γ and TNF-α production, both of which facilitate the activation of myeloid cells, such as macrophages, thus increasing phagocytosis and IFN-γ-inducing cytokine secretion. This results in a positive feedback loop that amplifies the infection-induced activation of NK cells and myeloid cells, leading to excessive inflammation, multiple organ failure, and increased mortality [60]. Indeed, Sherwood et al. demonstrated the deleterious effect of NK cells overactivation during sepsis using NK cell-depleted mice, which display decreased cytokine production, organ injury, and physiological dysfunction alongside increased resistance to mortality [67].

In addition to age-related phenotypic and functional alterations, most studies have shown that the number of NK cells is relatively stable or slightly higher in elderly individuals [68-71]. This slight increase is due to an elevated frequency of the more mature CD56^dim^ subsets, whereas the number of immature CD56^bright^ subsets is diminished in elderly individuals [72]. Functionally, NK cells exhibit normal or increased IFN-γ production but reduced cytotoxicity, which may be explained by a decrease in the release of perforin into the immunological synapse in elderly individuals [68, 73]. Few studies have investigated NK cell alterations in elderly septic patients due to their low numbers in peripheral blood [74]; however, we speculate that the increased production of IFN-γ by NK cells with age, which results in aggravated tissue damage, may explain why sepsis occurs more frequently in elderly patients.

IL-15 is an immune-adjuvant molecule that has demonstrated promise in the early stages of testing in clinically-relevant models of sepsis [75]. IL-15 is a pluripotent cytokine that is essential for NK cell development, survival, and function [76], whose stimulation has been shown to increase NK cell cytotoxicity and improve survival in sepsis [77, 78]. NK cells from both elderly and septic patients display reduced cytotoxicity, which may synergistically result in significantly reduced cytotoxicity in elderly septic patients, thus making these patients more susceptible to secondary infections or latent viral reactivation and leading to poorer outcomes. Consequently, IL-15 could be a promising therapeutic candidate for future trials in elderly septic patients.

### DCs

DCs are the most potent antigen-presenting cells and are key orchestrators of immune responses as they are critical for antigen uptake and presentation to naïve T cells and prime effective adaptive immune responses. During infection, DCs can be stimulated by pathogen-associated molecules via pattern recognition receptors on their surface [79]. Activated DCs then upregulate antigen-presenting (major histocompatibility complex (MHC)-I and II) and costimulatory (e.g. CD40, CD80, and CD86) molecules and proinflammatory cytokines (e.g. IFN-γ, TNF-α, IL-6, and IL-12), which enable them to effectively prime effector T cells [80]. DCs can be classified into two subsets: myeloid DCs, also known as conventional or classic DCs (cDCs), include mouse CD8α^+^ cDCs and CD11b cDCs, which have high antigen-presentation capacity and mainly produce proinflammatory cytokines, whereas plasmacytoid DCs (pDCs) are a lymphoid lineage that are the main source of type I interferons [81, 82].

Numerical and functional alterations in DCs have been widely reported in sepsis. For instance, the number of cDCs and pDCs in both the spleen and peripheral blood is substantially decreased, which may be induced by enhanced sepsis-related apoptosis [83, 84]. Consistently, Guisset et al. observed that this quantitative loss of DCs during sepsis was closely associated with severity and mortality [85], while the function of surviving DCs has been shown to decline for a long time after sepsis [86]. During sepsis, DCs exhibit reduced HLA-DR, CD80, and CD86 expression, resulting their inability to effectively present antigens and prime a robust effector T cell response [87, 88]. In addition, the expression of the B and T lymphocyte attenuator (BTLA) and CD155, which are known to inhibit T cell function, are increased in DCs during sepsis [89, 90]. Faivre et al. demonstrated that DCs isolated from septic patients are unable to induce robust effector T cell responses, instead inducing either T cell anergy or regulator T cell (Treg) proliferation [91, 92]. Furthermore, DCs produce fewer proinflammatory cytokines, such as IL-12 and TNF-α, and more anti-inflammatory cytokines, such as IL-10 and TGF-β [86, 91, 92]. Many mechanisms participate in DCs alterations during sepsis, including the induction of apoptosis, variation in Toll-like receptor-dependent signaling, activation of Wnt signaling, and epigenetic regulation [93]. Thus, DCs play a pivotal role in the development of sepsis-induced immunosuppression.

While the majority of studies have reported a reduction in the number of DCs, some studies have shown that the number of DCs is relatively stable or increases slightly in elderly individuals. Despite these conflicting studies regarding the number of DCs, the function of DCs has been shown to change significantly with age [94]. Agrawal et al. observed that DCs isolated from elderly individuals display a proinflammatory phenotype characterized by the increased basal secretion of proinflammatory cytokines, such as IL-6 and TNF-α, which may be closely related to the senescence-associated secretory phenotype, one of characteristics of immunosenescence [95-97]. In addition, Li et al. observed that CD8α^+^ DCs in elderly mice display poor upregulation of costimulatory molecules for MHC-II and CD40, which may produce a less effective T cell priming environment [98]. Furthermore, aged DCs have been shown to exhibit impaired migration, antigen uptake, pinocytosis, and phagocytosis [99]. Several mechanisms have been found to alter DC function during sepsis, including reduced AKT phosphorylation, mitochondrial dysfunction, histamine modification, reduced STAT1 and STAT3 protein phosphorylation, and defects in IκB-α signaling [95, 100-102].

Although there is a lack of studies investigating changes in DCs in elderly patients with sepsis, we speculate that the antigen-presenting ability of DCs is significantly lower in these patients due to the synergistic effects of age and sepsis, which decrease successful adaptive immune responses and increase susceptibility to secondary nosocomial infections.

As mentioned above, DCs from both elderly and septic patients display decreased number, HLA-DR expression, and function. Although DCs are prominent immune response orchestrators, there is a lack of immune modulatory therapies targeting DCs. FMS-like tyrosine kinase 3 ligand (FLT3L) is a DC growth factor that may have the potential to ameliorate sepsis-induced immunosuppression [103-105], while IL-15 may exert potent immune-stimulatory and proliferative effects against DCs [75]. Future studies should focus on reversing DC dysfunction in elderly patients with sepsis.

### ECs

ECs form a semi-permeable barrier in the vascular and lymphatic systems that is closely related to many physiopathological processes and participates in innate immune responses [28]. Due to their location, ECs are one of the first cell types to detect invading pathogens and act as danger signal sensors [106]. Activated ECs produce proinflammatory cytokines and chemokines that augment immune responses by recruiting immune cells after LPS exposure. ECs also express TLR-2 and TLR-4, which are crucial for the response to bacterial infection [107]. Moreover, their intact structure and function are highly important for maintaining appropriate circulation, balance between the coagulation and fibrinolysis systems, and avoiding microcirculatory disorders [108]. Thus, ECs are significant physiological and immunological regulators during infections.

Sepsis causes direct endothelial barrier destruction and macro- and microvascular endothelial dysfunction that manifests as a capillary leakage, altered vasomotor tone, and microvascular thrombosis, all of which lead to widespread tissue edema, disseminated intravascular coagulation, multiorgan dysfunction, and mortality [28]. Although many studies have shown that increased coagulation is associated with advanced age, little is known about the association between age and endothelial dysfunction in models of sepsis. Tucsek et al. compared the primary microvascular endothelial cells (MVECs) of young and old rats treated with serum from patients with sepsis and healthy controls. The aged septic MVECs displayed increased sensitivity to oxidative stress and cellular damage, suggesting that senescent ECs are more susceptible to sepsis-induced damage [109]. Similar susceptibility was demonstrated at the animal level by Wulfert et al., who showed that elderly septic mice exhibit high P-selectin, E-selectin, and PECAM-1 expression, which mediate leukocyte rolling, migration, and immunological cascades during the early stages of inflammation and are associated with uncontrolled inflammatory responses and high mortality [110, 111].

Therefore, age-associated and sepsis-induced EC dysfunction synergistically increase severity of dysfunction, which may suggest that elderly patients with sepsis are more susceptible to multiorgan dysfunction and high mortality.

## Numerical, phenotypic, and functional alterations in adaptive immune cells and potential therapeutic targets

Adaptive immune cells are specifically activated by pathogens or antigen-presenting cells and subsequently initiate potent anti-infection responses. In addition, these cells undergo vigorous proliferative expansion when re-encountering pathogens and provide increased protection following reinfection due to the formation of memory cell subgroups. Like innate immune cells, adaptive immune cells undergo significant alterations in number, phenotype, and function as a result of sepsis and aging.

### T cells

*γδ T cells:* The majority of T cells have T cell receptors (TCRs) composed of two α and β glycoprotein chains; however, γδ T cells, which account for just 5% of all T cells, have a TCR made up of one γ chain and one δ chain [28]. Approximately 5-10 % of γδ T cells are found in the peripheral blood, whereas they are more widespread within epithelial-rich tissues, such as the intestine, where they comprise up to 50% of all T cells [112]. γδ T cells play major roles as the first line of defense against pathogens in the mucosa. Although it remains unclear which antigens γδ T cells respond to, they are thought to recognize phosphorylated microbial metabolites and lipid-peptides from pathogens presented on mucosal surfaces, following which they mount a prompt, innate-like immune response by releasing IFN-γ, IL-17, and various chemokines [38, 113, 114]. In addition, γδ T cells can act as antigen-presenting cells to form a bridge between the innate and adaptive immune responses [115].

Studies have shown that septic mice and patients harbor significantly fewer γδ T cells, with more severe depletion accompanied by higher severity and mortality [115-117]. Galley et al. reported that γδ T cells in patients with sepsis are mainly CD27 negative, and act as nonproliferating cells, aggravating the loss of γδ T cells [118]. Furthermore, studies have shown that the function of γδ T cells is impaired during sepsis, with Liao et al. reporting that IFN-γ production is significantly impaired in the γδ T cells of septic patients and is closely associated with mortality [119]. To further explore their role in sepsis, γδ T cell-deficient mouse models were generated, which exhibited aggravated tissue damage, increased bacterial load, increased intestinal permeability, and decreased survival [117]. Thus, the decrease in γδ T cells number and function may be particularly detrimental to the host by making noninvasive intestinal pathogens invasive, thereby causing secondary infections following sepsis.

Studies have shown that age significantly affects γδ T cells, including their number, phenotype, and function. For instance, several studies have shown that elderly individuals have fewer γδ T cells under both baseline and infection conditions [120, 121], as well as significantly reduced proliferation which amplifies the decrease in number [122]. In addition to these numerical alterations, γδ T cells also undergo aging processes in a similar manner to αβ T cells, resulting in phenotypic and functional changes. For instance, aged γδ T cells appear to shift from an early (CD27^+^ CD28^+^ CD45RA^+^ CD16^-^) to a late (CD27^-^ CD28^-^ CD45RA^+^ CD16^-^) differentiated effector phenotype, thus reducing protection against new pathogens not only in peripheral blood, but also in mucosal tissues [120, 123].

Despite these findings, little research has been carried out regarding alterations in the γδ T cells of elderly septic patients, which should be the focus of further studies; however, it can be hypothesized that resistance to infection is weakened in elderly patients with sepsis due to numerical, phenotypic, and functional alterations in their γδ T cells.

*CD4^+^ T cells:* The most important peripheral lymphocytes are thought to be CD4^+^ T cells, which play a central role during anti-infection immunity by orchestrating effective immune responses and influencing both innate and adaptive immune cells via cytokine production and cell-to-cell interactions [124]. When they encounter peptide antigens presented by MHC-II molecules on antigen-presenting cells, CD4^+^ T cells activate, proliferate, and mount efficient immune responses. CD4^+^ T cells are also known as T helper (Th) cells, as they help other immune cells to prime protective immune responses; for instance, Th cells activate macrophages and neutrophils, initiate primary CD8^+^ T cell responses, and ensure efficient isotype switching in primary and memory B cell responses [125-127]. CD4^+^ T cells play important roles in many immunological responses as they can differentiate into various phenotypes following stimulation by different cytokines and costimulatory molecules, including the Th1, Th2, and Th17 cell subsets [128]. Th1 cells are induced by IL-12 and IFN-γ and produce IL-2 and IFN-γ in response to intracellular infections to provide the signals required for B cell isotype switching [129, 130]. In contrast, Th2 cells are activated by IL-4 to produce cytokines such as IL-4, IL-5, and IL-10, and play important roles in the response to helminthic infections and B cell activation [131, 132]. Th17 cells are induced by IL-6 in response to extracellular fungal and bacterial pathogens and produce IL-17 and TNF-α [133]. As such, loss or functional alterations in CD4^+^ T cells could have dramatic consequences for a broad variety of immune responses.

Numerous studies have investigated the effects of sepsis on CD4^+^T cells, including changes in their number, phenotype, and function. Sepsis increases apoptosis which can lead to deleterious defects in CD4^+^T cell number, with greater apoptosis associated with increased mortality [134]. In addition, sepsis has been shown to significantly reduce the proliferation capability of CD4^+^T cells, thus amplifying the decrease in their number [135]. With respect to phenotype and function, numerous studies have demonstrated markedly reduced cytokine release by both Th1 and Th2 cells and in their expression of transcription factors T-bet and GATA3, suggesting that sepsis suppresses both Th1 and Th2 cell functions. Similarity, a reduction in cytokine production by Th17 cells has also been observed, which may increase susceptibility to fungal infections [136, 137]. Studies of septic mice and patients have reported increases in the expression of coinhibitory receptors such as programmed cell death (PD-1), TNF-related apoptosis-inducing ligand (TRAIL), and B and T lymphocyte attenuator (BTLA) in CD4^+^ T cells, which partly explains their persistent reduced proliferative capacity and inflammatory cytokine production [138-140]. The mechanisms that participate in these alterations involve epigenetic reprogramming, such as histone methylation and chromatin remodeling, and metabolic reprogramming [141, 142], which result in CD4^+^ T cells with an anergic or exhausted profile that is closely related to an increased risk of secondary infections and higher mortality. In addition, the reduced function of antigen-presenting cells is also a key contributor toward CD4^+^ T cells dysfunction.

During aging, CD4^+^ T cells accumulate alterations that play an important role in immunosenescence. Indeed, the decrease in naïve CD4^+^ T cells and increase in memory CD4^+^ T cells could explain the age-related decline in their ability to respond to new antigens, which may be caused by thymic involution and lifelong chronic antigenic stimulation [143, 144]. During aging, the TCR repertoire declines and TCR signaling is reduced due to cytoskeletal rearrangement, cell surface glycosylation, and the phosphorylation of key signaling molecules such as tyrosine. Alongside telomere shortening and epigenetic reprogramming, these changes reduce the activation, expansion, and differentiation of aged CD4^+^ T cells, and reduces their ability to prime effective immune responses [145-148]. Moreover, Rink et al. observed that aged CD4^+^ T cells exhibit a shift in cytokine production from a Th1 to a Th2 phenotype [149].

Several studies have examined CD4^+^ T cells in elderly mice and patients with sepsis, revealing significant changes in their number, differentiation, and function. Both elderly mice and patients with sepsis display smaller CD4^+^ T cell populations as CD4^+^ T cells are more prone to undergo sepsis-induced apoptosis in elderly individuals, and cell proliferation is impaired [150, 151]. In addition, the balance between CD4^+^ T cells subgroups is disrupted in these individuals; for instance, Coakley et al. observed that CD4^+^ T cells expressing IL-23 receptor and RORγt are less common in elderly patients with sepsis, resulting in dysregulated Th17 responses [152]. In addition, Inoue et al. demonstrated increased PD-1 and CTLA-4 expression and decreased IL-2 production in CD4^+^ T cells in response to stimulation, resulting in more severe persistent CD4^+^ T cell exhaustion in both elderly mice and patients with sepsis [151]. Therefore, the reduced number, subgroup imbalance, and persistent exhaustion of CD4^+^ T cells is more severe in both elderly septic mice and patients, increasing susceptibility to secondary infections and mortality.

*CD8^+^ T cells:* CD8^+^ T cells are also known as cytotoxic T lymphocytes (Tc cells) and play crucial roles in the eradication of intracellular pathogens and tumor cells [153]. When they encounter peptide antigens presented by MHC-I molecules on antigen-presenting cells in the presence of costimulatory molecules, naïve CD8^+^ T cells are activated and undergo proliferative expansion [154]. The cells are then effectively characterized by their release of cytokines such as IFN-γ and TNF-α, the lysis of target cells via the secretion of perforin and granzyme B, or the induction of apoptosis via the Fas/FasL pathway [155, 156]. After the infection is resolved, only a small portion of effective CD8^+^ T cells survive to form a memory CD8^+^ T cell population that undergoes vigorous proliferative expansion when the pathogen is re-encountered to provide increased protection after reinfection [156, 157].

Numerous studies have demonstrated that sepsis induces substantial and long-lasting changes in CD8^+^ T cells, including their number, phenotype, and function. The number of naïve and memory CD8^+^ T cells is reduced by sepsis-induced apoptosis, as indicated by increased caspase 9, Bim, and Bid expression and decreased Bcl-2 expression in CD8^+^ T cells isolated from patients with sepsis [158, 159]. In addition, CD8^+^ T cells have been found to undergo phenotypic and functional alterations in mice and patients with sepsis. Condotta et al. observed an increase in the expression of coinhibitory molecules, such as PD-1, LAG-3, and 2B4, and a decrease in the release of effector cytokines such as IFN-γ, TNF-α, and IL-2, suggesting that CD8^+^ T cells are exhausted in mice and patients with sepsis [160-162]. Except for these intrinsic alterations, sepsis has also been shown to impair the function of Th1 cells, leading to CD8^+^ T cells dysfunction since Th1 cells play important roles in the activation of naïve CD8^+^ T cells and the formation of memory CD8^+^ T cells [163, 164]. Antigen-specific memory CD8^+^ T cells are responsible for immunosurveillance that protects against pathogens that are re-encountered and the reactivation of latent viral infections [165]. However, sepsis reduces the protective effects of these cells by decreasing their number and function [161]; therefore, the impact of sepsis on CD8^+^ T cells increases susceptibility to secondary infection and mortality.

Age-related changes in CD8^+^ T cells are also an important part of immunosenescence and are even more prominent than in CD4^+^ T cells. With age, the frequency of naïve CD8^+^ T cells decreases while that of terminally differentiated memory CD8^+^ T cells increases, which may be explained by thymus involution and a subsequent reduction in naïve CD8^+^ T cell output [166, 167], as demonstrated precisely by Shin et al. at the single-cell level [168]. In addition, Gustafson et al. observed a decline in IL-7R expression in naïve CD8^+^ T cells with age which directly correlated with a reduction in their frequency [169], while Qi et al. observed a modest decrease in TCR repertoire diversity alongside reduced TCR signal transduction in CD8^+^ T cells [170]. Moreover, reduced effector molecule production and impaired cytolytic activity have been reported in aged CD8^+^ T cells [171]. As well as these intrinsic changes in CD8^+^ T cells due to aging, defects in the priming environment that support the initiation of CD8^+^ T cell responses also play a significant role. For instance, antigen-presenting cells such as DCs display impaired maturation, reduced antigen uptakes, and reduced CD80/CD86 expression during aging, which negatively affect CD8^+^ T cell priming [172].

Although CD8^+^ T cells have been studied extensively in both septic and aging patients, few studies have specifically examined CD8^+^ T cells in elderly patients and animals with sepsis. Saito et al. observed that the CD8^+^ T cell population is smaller in elderly septic mice than in their younger counterparts due to increased susceptibility to sepsis-induced apoptosis. Moreover, CD8^+^ T cells expressing PD-1 were more common in elderly septic mice, which may result in more severe persistent CD8^+^ T cell exhaustion and increased susceptibility to latent virus reactivation [150]. Further studies are required to clarify the numerical, phenotypic, and functional alterations in CD8^+^ T cells and their underlying mechanisms in elderly septic patients.

*Tregs:* Tregs are CD4^+^ CD25^+^ CD127^low^ FOXP3^+^ T cells, also known as suppressor T cells that are a subset of CD4^+^ T cells induced by IL-10 and produce IL-10 and TGF-β [173-175]. Despite making up only a small fraction of T lymphocytes, Tregs are specialized immune cells that repress the functions of both adaptive and innate immune cells and play a significant role in maintaining self-tolerance, suppressing autoimmune diseases and sustaining immune homeostasis under pathological conditions [176].

Increased Treg percentage has been reported in both animals and patients with sepsis, and the persistence of this increase is closely related to increased mortality [177]. Remarkably, both the relative and absolute number of Tregs increases, which has been attributed to various possible reasons. Firstly, Tregs are more resistant to sepsis-induced apoptosis, possibly because they display higher expression of Bcl-2, an anti-apoptosis protein, than other effector T cells [38]. Secondly, an increase in the level of heat shock proteins and histones is likely to increase the number of Tregs via TLR2 signaling during sepsis [178]. Thirdly, increases in both protein and mRNA levels of Foxp3, which was identified as the master gene for Treg differentiation and development, was shown to be partly involved in JNK/AP-1 activation [179, 180]. Fourthly, endogenous IL-33 released in response to severe tissue damage has been shown to activate type 2 innate lymphoid cells to produce IL-4 and IL-13, which drive M2 macrophage polarization and result in Treg cell expansion via IL-10 production [181]. Alongside the increased numbers of Tregs during sepsis, their suppressive effects are also amplified. For instance, it has been reported that Tregs participate in the loss of monocytes in septic patients via a proapoptotic mechanism involving the Fas/FasL pathway [182], while Tregs also significantly reduce the ability of monocytes/macrophages to respond to LPS during sepsis [183]. Moreover, Tregs precipitate in an NK cell-dependent endotoxin tolerance-like phenomenon characterized by decreased IFN-γ production and can also suppress adaptive immune cells by inhibiting effector T cell proliferation and function, which can be abrogated by Foxp3-specific siRNA [180,184]. Consequently, increases in Treg number and their suppressive effects negatively impact both innate and adaptive immune cells, thus contributing toward increased susceptibility to nosocomial infections and higher mortality.

Tregs also display age-related changes in frequency and function; for instance, substantial evidence has revealed that Treg frequency increases with age in both humans and mice, and is associated with age-related hypomethylation of the upstream Foxp3 enhancer, which results in increased Foxp3 expression [185-187]. Consistently, Chougnet et al. observed that the expression of the proapoptotic factor Bim decreased in the Tregs of aged mice, causing Treg accumulation with age by reducing apoptosis [188]. In terms of functionality, Tregs isolated from aged mice exert greater suppressive effects than those from younger mice by producing more IL-10, downregulating CD86 expression on DCs, and suppressing effector T-cell proliferation [187]. Therefore, enhanced Treg frequency and function may induce immunosenescence and contribute toward decreased immunity against pathogens and tumor cells in elderly individuals.

Despite the lack of research into Treg alterations in elderly septic patients, we speculate that both aging and sepsis exert additive effects to increase Treg frequency and suppressive function, thus reducing the immunity of elderly septic patients.

Since the number and function of CD4^+^T and CD8^+^T cells decrease in elderly patients with sepsis, while Tregs increase in frequency and suppressive function in both aging and septic patients, IL-7 administration could a possible treatment strategy. IL-7 is a hematopoietic cytokine produced by stromal cells that is crucial for the production, development, homeostasis, maintenance, and effector functions of T cells, but negatively regulates Tregs [189-191]. Recombinant IL-7 is thought to improve multiple functional aspects of T cells, such as inhibiting the apoptosis of peripheral T cells via upregulating Bcl-2 expression, increasing TCR diversity which is lost in elderly septic patients, and increasing the expression of cell adhesion molecules to result in better cell trafficking to sites of infection [192, 193]. In addition, IL-7 treatment has been reported to reduce T cell apoptosis, restore IFN-γ production, facilitate pathogen clearance, and improve survival in mouse models of sepsis [193, 194]. Similarity, *ex vivo* experiments using cells from septic patients have shown that IL-7 treatment significantly improves T cell functions such as proliferation, IFN-γ production, and Bcl-2 induction [195]. Since IL-7 is also hypothesized to rejuvenate immune functions in elderly individuals [196], clinical trials of IL-7 in elderly septic patients are highly anticipated. T cells from these patients also display increased expression of negative costimulatory molecules, such as PD-1 and PD-L1, which produce inhibitory signals that reduce T cell proliferation and function, and are closely associated with T cell exhaustion [197]. Since both anti-PD-1 and anti-PD-L1 therapies have demonstrated promising results in human trials involving cancer and viral infection [28, 198], they may also have similar beneficial results in elderly septic patients by reversing T-cell dysfunction. In mouse models of sepsis, anti-PD-1 and anti-PD-L1 antibody treatments have been shown to increase pathogen clearance, restore protective immune responses, and improve survival [199-201], while *ex vivo* experiments using cells from patients with sepsis have revealed that PD-1 or PD-L1 pathway blockade decreases sepsis-induced immune dysfunction [202]. Considering their ability to reverse T cell dysfunction, anti-PD-1 and anti-PD-L1 antibodies could be promising therapies for improving immune function and long-term survival in elderly septic patients, as well as similar therapeutic strategies targeting other negative costimulatory molecules expressed on T cells, such as anti-CTLA4, anti-Tim-3, and anti-LAG-3 antibodies.

### B cells

B lymphocytes are an adaptive immune cell lineage of multifunctional cells generated from bone marrow that play significant roles in both innate and adaptive immune responses. B cells recognize a wide variety of antigens, such as proteins, lipids, polysaccharides, nucleic acids, and chemicals, depending on the type of antigen that specifically binds to B cell receptor (BCR) with or without the assistance of Th cells [203, 204]. In the presence of costimulatory molecules, naïve B cells activate, proliferate, and differentiate into plasma cells that secrete effector antibodies. After the infection has been resolved, only a small portion of effector B cells survive to constitute a population of memory B cells, which undergoes vigorous proliferative expansion when the pathogen is re-encountered [126]. Although B cells are mostly known for their role in humoral immune responses via specific immunoglobulin secretion and some degree of isotype switching, they also act as antigen-presenting cells to activate T cells via MHC-II expression [205, 206].

Due to their multifunctionality, B cells play pivotal roles in many immune processes, including sepsis. Kelly-Scumpia et al. demonstrated that B cell-deficient mice exhibit higher mortality during sepsis [207], while several studies have described alterations in B lymphocyte number, phenotype, and function during sepsis. Although fewer studies have investigated B lymphocytes than T lymphocytes, it has been reported that the number of B cells decreases significantly during sepsis [208]. This progressive depletion is greater in memory B cells than in naïve B cells, suggesting that the reduction in number is not the result of impaired bone marrow generation but sepsis-induced apoptosis, which may be associated with higher phosphorylation of ERK, an intracellular kinase related to transition to apoptosis, and increased expression of CD95, a cell-death receptor [158, 209]. Impaired B cell maturation also contributes toward reduced B cell numbers due to the loss of antigen-presenting cells and Th cells during sepsis [210, 211]. The phenotype and function of circulating B cells has also been shown to change markedly during sepsis, with Gustave et al. observing a shift toward an exhausted-like phenotype (CD21^low^ CD95^high^), an increased proportion of regulatory B cells (Bregs) producing a significant amount of IL-10. These phenotypes are consistent with the reduced activation and proliferation capacities of B cells, reduced antibody secretion, and reduced antigen-presenting capacity due to decreased MHC-II expression during sepsis [210, 212-214].

Alterations in B cells have also been reported with age, including a decline in their number associated with decreased hemopoietic stem cell function in an aged bone marrow microenvironment [215-217]. In addition, the blood plasma levels of BAFF and APRIL, which are important survival factors for mature peripheral B cells, are reduced in elderly individuals and contribute toward a decreased number of B cells [218]. Similarity, B cells function is known to decline markedly with age, leading to less robust humoral immune responses in elderly individuals and a reduction in the protective effects of the immunoglobulins produced by B cells due to their low titer and affinity as a result of intrinsic and extrinsic defects [144, 219]. These intrinsic defects include a progressive decline in germinal center formation, which leads to decreased somatic hypermutation and antibody affinity maturation. Similarity, age-related decreases in activation-induced cytidine deaminase (AID) expression due to E47 downregulation decreases class switch recombination and affinity maturation in elderly individuals [220]. The reduced expression of costimulatory molecules, such as CD86, and defective BCR signaling due to reduced diversity may also play a role [221], while the reduction in the number of naïve B cells has been shown to be accompanied by memory B cell expansion, suggesting an impaired ability to produce high-affinity protective antibodies against newly encountered antigens [1]. On the other hand, extrinsic defects refer to the age-related dysfunction of CD4^+^ helper cells, which are required for germinal center formation and the activation of somatic mutation genes, but become less able to help produce high-affinity antibodies with age [222-224]. Together, the age-related numerical and functional alterations in B cells result in poor humoral immune responses in elderly individuals.

Unfortunately, few studies have investigated B cells alterations in elderly septic patients. Suzuki et al. observed impaired IgM production and decreased serum IgM in elderly septic patients, which may increase susceptibility to secondary infections, particularly in elderly individuals [225]; however, further studies are required. Aging and sepsis related effects on various elements of the adaptive and innate immune systems are summarized in Table 1.

Although B cells play significant roles in both innate and adaptive immune responses, few studies have focused on immune modulatory therapies targeting B cells. Padgett et al. reported that androstenediol, a dehydroepiandrosterone (DHEA) metabolite, was able to improve B cell responses in an experimental mouse model of aging [226]; however, further research is required to improve humoral immune responses by reversing decreases in B cell number and function in elderly patients with sepsis.

**Table 1:** Defects of immune cells with aging and sepsis.

Immune component	Alterations associated with sepsis	Alterations associated with aging
Innate immune cells		
Neutrophils	delayed apoptosis; increased immature CD10^dim^ CD16^dim^ proportion; impaired production of bactericidal agents; reduced chemotactic response	preserved number; impaired chemotaxis and phagocytosis; impaired the ability to form NETs
Monocytes and macrophages	reduced HLA-DR expression; shift to immunosuppressive M2 cell type; PD-L1 overexpression; reduced pro-inflammatory cytokine production; increased anti-inflammatory mediator production; impaired antigen presentation	constant number; reduced HLA-DR expression; less pro-inflammatory cytokines production; impaired phagocytosis and efferocytosis; impaired antigen presentation
Natural killer cells	decreased number; increased apoptosis; lower expression of NKG2D; reduced IFN-γ secretion; impaired cytotoxicity	stable or slightly increased number; increased mature CD56^dim^ subsets; decreased immature CD56^bright^ subsets; normal or increased IFN-γ production; reduced cytotoxicity
Dendritic cells	decreased number; increased apoptosis; reduced HLA-DR and CD80 and CD86 expression; increased BTLA and CD155 expression; decreased pro-inflammatory cytokine production, increased anti-inflammatory cytokine production; impaired antigen presentation	decreased number; poor upregulation of HLA-DR and CD40; increased pro-inflammatory cytokine production; impaired migration; impaired antigen presentation; impaired pinocytosis and phagocytosis
Adaptive Immune Cells		
γδ T cells	decreased number; mainly CD27^negative^; impaired IFN-γ production	decreased number; shift from an early to late-stage differentiated phenotype; reduced proliferation; reduced responses to pathogens
CD4^+^ T cells	decreased number; increased apoptosis; display an anergic or exhausted profile; increased PD-1 and TRAIL and BTLA expression; reduced production of cytokine released by Th1, Th2, and Th17 cells; reduced proliferation	loss of naïve CD4^+^T cells; expansion of memory CD4^+^ T cells; declined TCR repertoire; shift from Th1 to Th2 cytokines; declined responses to new antigens; reduced capacity to be activated, expanded and differentiated
CD8^+^ T cells	decreased number; display an exhausted phenotype; increased PD-1, LAG-3, and 2B4 expression; reduced IFN-γ, TNF-α, and IL-2 production	loss of naïve CD8^+^T cells; expansion of terminal differentiated memory CD8^+^T cells; declined TCR repertoire diversity; reduced effector molecules production; impaired cytotoxic activity
Tregs	increased percentage and number; increased Foxp3 expression; amplified suppressive effect	increased number; higher expression of Foxp3; amplified suppressive effect
B cells	decreased number (especially memory B cells); increased apoptosis; display an exhausted phenotype; increased Bregs proportion; increased IL-10 production; impaired maturation; reduced activation and proliferation capacities; less antibody secretion; impaired antigen presentation	decreased number; loss of naïve B cells; expansion of memory B cells; reduced CD86 expression; reduced BCR repertoire diversity; low titer and affinity immunoglobulins

## Potential mechanisms underlying immune dysfunction in the elderly with sepsis

Immune dysfunction is much more significant in elderly patients with sepsis than in their younger counterparts and several potential underlying mechanisms have been proposed. Stable cell number is known to play an important role in maintaining cellular function; therefore, the loss of most immune cells in elderly patients with sepsis due to age-associated degeneration of both central and peripheral immune organs will at least partly result in immune dysfunction. In addition, there is evidence of synergism between aging and sepsis in the promotion of apoptosis, potentially due to mitochondrial dysfunction [227, 228]. For instance, Escames et al. observed that both aging and sepsis increase the mitochondrial production of nitric oxide and free radicals to stimulate mitochondrial nitric oxide synthase [228], which then inhibits the mitochondrial respiratory chain and worsens mitochondrial damage, leading to more severe cellular apoptosis [228]. Mitochondrial dysfunction can also directly affect ATP generation, which is necessary for immune cell function; thus, severe lack of ATP could trigger immune cell anergy [229]. In addition, cellular metabolism has emerged as a key mechanism regulating inflammatory responses [230]. Although this mechanism appears complex, imbalance between cellular metabolic processes may play crucial roles in immunoparalysis in elderly patients with sepsis and thus represents an attractive new therapeutic approach [231]. Furthermore, perturbations in the epigenetic regulation of gene function, such as DNA methylation, histone modification, and chromatin remodeling, may also underlie immune dysfunction in the elderly septic patients and contribute toward the immunosuppressive phenotype of immune cells [232, 233]. Despite these promising findings, a considerable amount of work is required to fully elucidate the mechanisms underlying immune dysfunction in the elderly with sepsis.

## Conclusions

Elderly individuals display increased susceptibility and mortality to sepsis, which places an increased burden on global medical resources. Aging is associated with peculiar immune responses to sepsis involving immunosenescence, which causes complicated changes in each cell type of the innate and adaptive immune systems. These immune cells not only display different functions themselves, but also interact to form large regulatory networks. These immune cell alterations in elderly septic patients are largely responsible for their poor prognosis and increased mortality; thus, research on the specific alterations in each innate and adaptive immune cell type in elderly mice and patients with sepsis is urgently needed. Furthermore, early recognition and appropriate combined immune modulatory therapies targeting immune cells are key to the successful management of sepsis in elderly individuals.
